# Affinity Maturation of Monoclonal Antibody 1E11 by Targeted Randomization in CDR3 Regions Optimizes Therapeutic Antibody Targeting of HER2-Positive Gastric Cancer

**DOI:** 10.1371/journal.pone.0134600

**Published:** 2015-07-30

**Authors:** Bong-Kook Ko, Soyoung Choi, Lei Guang Cui, Young-Ha Lee, In-Sik Hwang, Kyu-Tae Kim, Hyunbo Shim, Jong-Seo Lee

**Affiliations:** 1 Therapeutic antibody research center, AbClon Inc., Seoul, Korea; 2 Departments of Bioinspired Science and Life Science, Ewha Womans University, Seoul, Korea; National Cancer Institute, NIH, UNITED STATES

## Abstract

Anti-HER2 murine monoclonal antibody 1E11 has strong and synergistic anti-tumor activity in HER2-overexpressing gastric cancer cells when used in combination with trastuzumab. We presently optimized this antibody for human therapeutics. First, the complementarity determining regions (CDRs) of the murine antibody were grafted onto human germline immunoglobulin variable genes. No difference in affinity and biological activity was observed between chimeric 1E11 (ch1E11) and humanized 1E11 (hz1E11). Next, affinity maturation of hz1E11 was performed by the randomization of CDR-L3 and H3 residues followed by stringent biopanning selection. Milder selection pressure favored the selection of more diverse clones, whereas higher selection stringency resulted in the convergence of the panning output to a smaller number of clones with improved affinity. Clone 1A12 had four amino acid substitutions in CDR-L3, and showed a 10-fold increase in affinity compared to the parental clone and increased potency in an *in vitro* anti-proliferative activity assay with HER2-overepxressing gastric cancer cells. Clone 1A12 inhibited tumor growth of NCI-N87 xenograft model with similar efficacy to trastuzumab alone, and the combination treatment of 1A12 and trastuzumab completely removed the established tumors. These results suggest that humanized and affinity matured monoclonal antibody 1A12 is a highly optimized molecule for future therapeutic development against HER2-positive tumors.

## Introduction

Monoclonal antibodies are mainstream treatments in oncology and autoimmune diseases, and are expected to play important roles in the future of disease treatment [1, 2]. More than 30 recombinant antibodies are currently approved by the United States Food and Drug Administration, of which approximately half are anti-cancer antibodies. Gastric cancer is one of the most common cancers and is the third leading cause of cancer death worldwide [3]. In gastric cancer, overexpression of epidermal growth factor receptor (EGFR), human epidermal growth factor receptor 2 (HER2), and HER3 is correlated with poor prognosis [4, 5]. Recently, the HER2 targeting monoclonal antibody trastuzumab was approved for treatment of HER2-positive metastatic gastric and gastroesophageal junction cancer based on results of the Trastuzumab with chemotherapy in HER2-positive advanced Gastric Cancer (ToGA) clinical trial [6].

Particular combinations of mutually noncompetitive antibodies targeting the same receptor increase anti-tumor activity *in vitro* and *in vivo*. Combination of the HER2 targeting antibodies, trastuzumab and pertuzumab, shows increased efficacy in HER2-overexpressing breast cancers [7]. The benefits of the pertuzumab and trastuzumab combination have been further demonstrated in preclinical and clinical trials [8, 9]. Other HER2-targeting antibodies showing better efficacy in combination than as single agents, and have shown consistent down-regulation of HER2 levels and beneficial combination effects in mouse models [10]. The increased efficacy of antibody combinations has also been demonstrated with EGFR-targeting antibodies [11, 12] and vascular endothelial growth factor receptor 3 (VEGFR3)-targeting antibodies [13].

Therapeutic antibodies typically are extensively engineered to possess desirable biological and physicochemical properties, such as low immunogenicity, high affinity and specificity, optimal effector functions, and good solubility and stability [14]. Especially, antibody humanization and affinity maturation are two of the most frequently applied engineering processes during the development of therapeutic antibody candidates. Immunogenicity of therapeutic antibody limits the clinical utility and efficacy by production of anti-drug antibodies [15], and the humanization of the antibodies from mouse or other species is now a standard procedure for the development of therapeutic antibodies. A few humanization methods have been developed that employ either rational or empirical approaches [16]. The complementarity-determining region (CDR) grafting approach as a method to overcome the human anti-chimeric antibody (HACA) response [17] is a well-established humanization method. However, direct grafting of murine CDRs onto a human framework acceptor sequence often results in a loss of affinity, so back-mutations of framework region residues (Vernier zone residues) supporting the structure of CDR loops is often necessary [18]. For *in vitro* affinity maturation, three diversification approaches are typically used: random mutagenesis by e.g. error-prone PCR, randomization of targeted residues using degenerate oligonucleotides, and chain shuffling. In the targeted randomization approach, CDRs are the logical target for the randomization in most cases because somatic hypermutation has evolved to favor mutations in CDRs of antibodies [19], and CDR-H3 and CDR-L3 tend to dominate the antibody-antigen interaction [20]. One of the main problems associated with the targeted randomization is selecting the positions that are not essential for the antigen binding, but which can enhance the affinity when optimal substitution of amino acid is made. Alanine scanning can help determine the residues to randomize, especially when CDRs are long. Sometimes, alanine mutation itself increases the affinity of antibodies [21].

We previously developed a murine antibody targeting HER2 (clone 1E11) that shows synergistic antitumor activity in combination with trastuzumab in HER2 overexpressing gastric cancer cell lines [22]. In this report, we describe how we optimized the 1E11 for a therapeutic antibody by CDR grafting to human germline immunoglobulin variable genes and affinity maturation through targeted randomization of CDR-H3 and CDR-L3. The optimized 1E11 antibody (clone 1A12) shows synergistic antitumor activity in HER2-positive gastric cancer xenograft models in combination with trastuzumab. It was observed that for the clone 1E11, human germline variable genes are suitable acceptors for humanization without affinity reduction, and the substitution of CDR-L3 residues that are not essential for antigen binding was enough to improve the affinity by more than 10-fold.

## Materials and Methods

### Cell lines and materials

NCI-N87 cells were purchased from American Type Culture Collection (ATCC, Manassas, VA, USA) and OE-19 cells were obtained from the European Collection of Cell Culture (ECACC, Porton Down, UK). The cell culture medium was RPMI-1640 supplemented with 10% fetal bovine serum (FBS), and antibiotics and cells were cultured at 37°C under 5% CO_2_. Trastuzumab and palivizumab was produced by Genentech (South San Francisco, CA, USA) and MedImmune, LLC (Gaithersburg, MD, USA), respectively. ChromPure human IgG (Jackson ImmunoResearch, West Grove, PA, USA) was used as human IgG control antibody for *in vitro* assays. IgG antibodies were produced using the Freestyle 293 system (Invitrogen, Carlsbad, CA, USA) and purified using protein-A affinity chromatography (GE Healthcare, Piscataway, NJ, USA). Endotoxin was removed with an Endotoxin Removal Kit (GenScript, Piscataway, NJ, USA), and endotoxin levels were determined using an Endotoxin Detection Kit (GenScript). Recombinant proteins were produced as secreted proteins using the Freestyle 293 system and purified using protein-A or Ni-NTA chromatography (Qiagen, Valencia, CA, USA) for Fc-tagged or His-tagged proteins, respectively.

### Alanine-scanning mutagenesis and Fab purification

Site-directed mutagenesis for alanine scanning was performed by PCR mutagenesis using QuickChange Site-Directed Mutagenesis Kit (Agilent Technologies, Santa Clara, CA, USA). Mutant Fab proteins were expressed and purified to evaluate the importance of each residue for antigen binding activity. In brief, *Escherichia coli* DH5α cells transformed with the pComb3X vector harboring mutant Fab genes were grown at 28°C in SB broth. Expression was induced with 1 mM isopropyl β-D-1-thiogalactopyranoside when the optical density (600 nm) of the culture reached 0.8. Cell pellets were resuspended in chilled extraction buffer (120 mM Tris, pH 8.0, 0.3 mM EDTA, and 300 mM sucrose) and incubated on ice for 30 minutes for periplasmic extraction. Magnesium chloride (2.5 mM) was added to the clarified extract after centrifugation to scavenge free EDTA prior to immobilized metal ion affinity chromatography (IMAC) purification. Purified Fab proteins were used for ELISA binding assay and *k*_off_ analysis using surface plasmon resonance (SPR).

### Affinity maturation

Humanized 1E11 cloned in pComb3X vector as Fab format was utilized as the template for overlap extension polymerase chain reaction (PCR) mutagenesis as described earlier [23]. The degenerate oligonucleotides (Integrated DNA Technologies, Coralville, IA, USA) 5’-CTGCCCGAAGGTCCAGGGMNNMNNMNNMNNCTGCTGGCAGTAATAAGTAGC-3’ (reverse) and 5’-GTCTACTATTGTGCTAGA42S43S11S11S24S14S34STTCGACTACTGGGGCCAGGG-3; (forward) were used for L-NNK and H-XXS library, respectively. N denotes A, C, G or T; M is C or A; and S is G or C. Numbered base positions indicate hand-mixed nucleotides composed of 70% of one base and 10% each of the other three bases: 70% frequency base is G, A, T, and C for 1, 2, 3, and 4, respectively. For the H-2AA library, an equimolar mixture of the following forward mutagenic oligonucleotides were used: 5’-GTCTACTATTGTGCTAGANNKNNKGGTGGGACCGCCTCCTTCGAC-3’, 5’-GTCTACTATTGTGCTAGACACNNKNNKGGGACCGCCTCCTTCGACTAC-3’, 5’-GTCTACTATTGTGCTAGACACCTGNNKNNKACCGCCTCCTTCGACTACTGG-3’, 5’-GTCTACTATTGTGCTAGACACCTGGGTNNKNNKGCCTCCTTCGACTACTGGGGC-3’, 5’-GTCTACTATTGTGCTAGACACCTGGGTGGGNNKNNKTCCTTCGACTACTGGGGCCAG-3’, and 5’-GTCTACTATTGTGCTAGACACCTGGGTGGGACCNNKNNKTTCGACTAC TGGGGCCAGGG-3’. K denotes G or T. After two rounds of overlap extension PCR, Fab library DNA with a randomized CDR was cut with SfiI, ligated into the SfiI-digested phagemid vector pComb3X, and electroporated into *E*. *coli* strain ER2537 (New England Biolabs, Beverly, MA, USA).

High affinity binders were selected using soluble biotinylated HER2-ECD protein. Bead binders were pre-depleted by incubating the antibody phage library with 100 μL of pre-blocked Dynabeads M-280 Streptavidin (Invitrogen) for 1 hour with slow rotation at room temperature. Biotinylated HER2-ECD protein (100 nM– 0.1 pM) was added to the pre-depleted antibody library and incubated for 1 hour with rotation at room temperature. Then, 50 μL of blocked streptavidin-magnetic beads were added and incubated on the rotator for 15 minutes. Beads were washed 10 times with 1 mL of Tris-buffered saline-Tween 20 (TBST) and twice with 1 mL of PBS. Bound phages were eluted by incubating the beads with 1 mL of 100 mM triethylamine for 8 minutes then neutralized with 0.5 mL of 1 M Tris, pH 7.4.

### ELISA binding activity test

Antibodies were added to Costar 96-well half area plates (Corning, Corning, NY, USA Product No. 3690) coated with 1 μg/mL of the antigen. After incubation at room temperature for 1 hour, the plates were washed three times with TBST and incubated with rabbit anti-human antibody-horseradish peroxidase (HRP) (Pierce, Rockford, IL, USA) for IgG antibodies and rat anti-HA-HRP (Clone F10, Roche Life Science, Basel, Switzerland) for Fab antibodies, respectively. Plates were washed three times, tetramethylbenzidine (TMB) peroxidase substrate (SurModics, Eden Prairie, MN, USA) was added and reactions were stopped by adding 1 N sulfuric acid (DukSan, Ansan, Korea). Absorbance at 450 nm was measured using a Victor X3 instrument (PerkinElmer, Waltham, MA, USA).

### Surface plasmon resonance analysis

For *k*_off_ analysis of Fab proteins, HER2-ECD-His protein was immobilized onto the surface of a CM5 sensor chip (GE Healthcare) using the amine coupling method at approximately 2,000 response units (RU). Purified Fab proteins were injected at 2 μg/mL concentration and a flow rate of 50 μL/minute. For *k*_off_ analysis of IgG antibodies, goat anti-human IgG (γ) (Invitrogen, Cat. No. H10500) was immobilized onto the CM5 sensor chip using amine coupling, and antibodies were captured at 1 μg/mL for 4 minutes and stabilized for 5 minutes at a flow rate of 50 μL/minute. HER2-ECD-His protein was injected at a concentration of 160 nM. For affinity measurements, antibodies were captured at approximately 50 RU by goat anti-human IgG (γ) immobilized on a CM5 chip. HER2-ECD-His protein was injected at concentrations ranging from 0 to 640 nM. Sensorgrams were obtained at each concentration and evaluated using the BIAevaluation software. For epitope binning, IgG form of hz1E11 was immobilized onto separate CM5 sensor chip surfaces at approximately 1000 response units. HER2-ECD-His (320 nM) and antibodies (1 μg/mL) were sequentially added for 4 minutes and stabilized for 5 minutes at a flow rate of 50 μL/minute.

### Cell viability assay

Cells were seeded in 96-well plates (Corning) in the 10% FBS containing medium and pre-cultured for 24 hours. The cells were treated with antibodies at the indicated concentrations and culture for 4 days. WST-1 reagent (DoGen EZ-Cytox; Daeil Lab Service, Seoul, Korea) was used to measure cell viability. Relative cell viability was calculated by dividing the absorbance of each well by the mean absorbance of PBS-treated wells in each plate.

### Xenograft study

Athymic nude female mice (Daehan Biolink, Chungbuk, Korea) were injected subcutaneously in the left flank area with 5 × 10^6^ of NCI-N87 cells in Matrigel (BD Biosciences, San Jose, CA, USA). Tumors were allowed to grow to approximately 200 mm^3^ in size, and mice were then randomized into groups. Animals received intraperitoneal administration of antibodies at the indicated doses twice weekly. Tumor volumes were calculated using the formula (L×W×W)/2, where L represents the largest tumor diameter and W represents the smallest tumor diameter. Two-way repeated measures ANOVA followed by Bonferroni post test was performed for the statistical analysis of the tumor growth.All animal studies were conducted in accordance with the guidelines of the NIH’s “Guide for Care and Use of Animals” and an approved protocol received by the company’s Institution Animal Care and Use Committee.

## Results

### Humanization of 1E11 conducted by CDR-grafting to human germline genes

**Fig 1:**
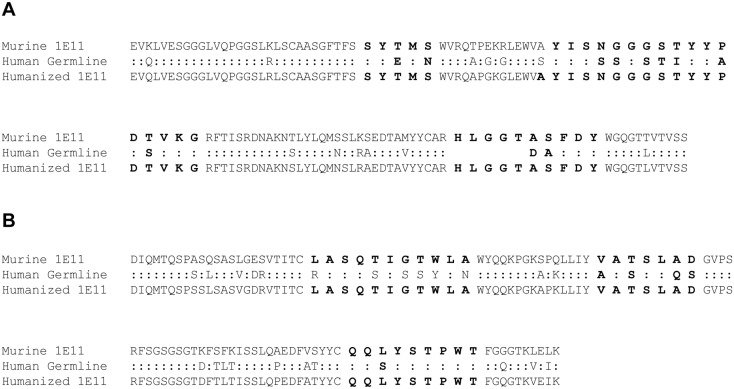
Humanization of 1E11 by CDR grafting. Heavy chain (**A**) and light chain (**B**) amino acid sequence alignment of murine, human germline, and humanized 1E11. The CDR residues, defined according to the Kabat definition, are shown in bold. Amino acids are numbered according to the Kabat numbering scheme. Colons represent common residues between murine and human germline sequences.

To develop the humanized antibody, the VH and VL sequences of the murine 1E11 were compared with human germline V and J gene repertories using IMGT/V-QUEST analysis tools [24]. For the heavy chain, IGHV3-48*03 and IGHJ4*01 exhibited the highest homology to the 1E11 counterparts, sharing 85% and 87% identity, respectively. For the light chain, human IGKV1-39*01 and IGKJ1*01 genes displayed identity of 80% and 81%, respectively. These human genes were selected as acceptor sequences for the grafting of the murine CDRs. Among the Vernier zone, which consists of residues in the framework region that are involved in the presentation of CDR structures by supporting the CDR loops [18, 25], only one residue at position 49_H_ of heavy chain differed between murine and human sequences. Consequently, humanized 1E11, hz1E11, has only one murine residue in the framework regions (Fig 1).

**Fig 2:**
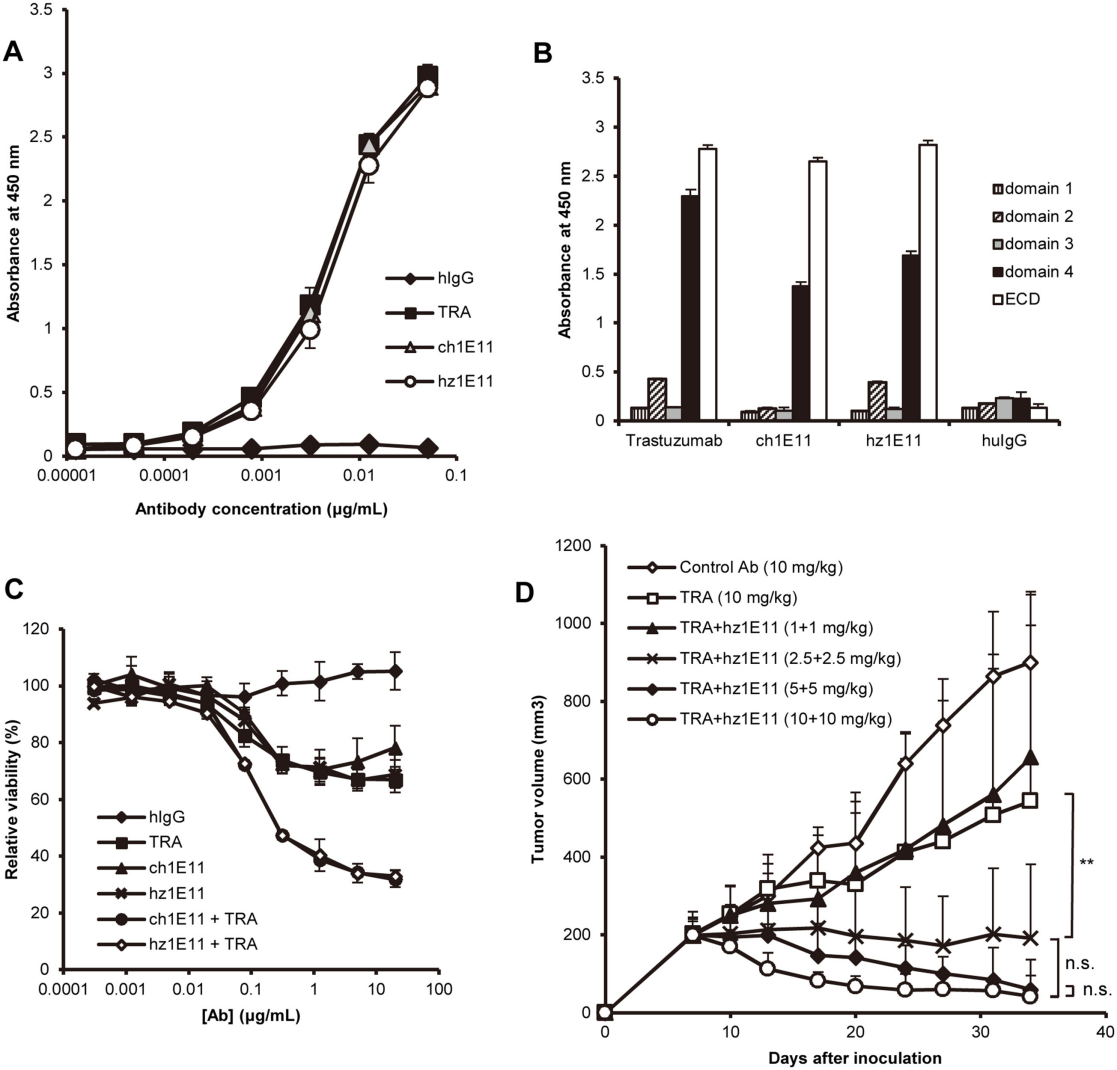
Humanized 1E11 shows equivalent binding properties and biological activity to the parental murine antibody. **A**, The binding activities of hz1E11 and ch1E11 to HER2-ECD protein were analyzed by ELISA. Trastuzumab (TRA) was used as a positive control antibody against HER2 protein. **B**, The binding activity of the antibodies was analyzed by ELISA using recombinant HER2 sub-domain proteins. **C**, NCI-N87 cells were treated with antibodies for 4 days in the complete growth media and the cell viability was measured in duplicates (mean ± SD) using WST-1 reagent. The 100% viability was defined as the viability of the antibody-untreated wells. **D**, Mice bearing NCI-N87 xenograft tumors were treated with indicated dose of control antibody, trastuzumab, hz1E11, or trastuzumab plus hz1E11. Palivizumab was used as the isotype control antibody. Tumor volume (mm^3^) was expressed as mean ± SD (*n* = 6 mice/group). For clarity, only positive error bars are shown. **, *P* < 0.01; n.s., not significant as determined by two-way repeated measures ANOVA followed by Bonferroni post test.

Binding activity of hz1E11 to extracellular region (ECD) of HER2 was equivalent to that of ch1E11 (Fig 2A), and the affinity of trastuzumab, ch1E11, and hz1E11 was 3 nM, 23 nM, and 23 nM, respectively. We also confirmed that hz1E11 bound to sub-domain IV like the parental ch1E11. Trastuzumab also binds to sub-domain IV [26]. The *in vitro* anti-proliferative activities of hz1E11 as a single agent and in combination with trastuzumab were also equivalent to those of ch1E11 (Fig 2C). In the previous study [22] it was reported that ch1E11 had *in vivo* antitumor activity comparable to that of trastuzumab as a single agent, and the combination of ch1E11 and trastuzumab resulted in the tumor regression index (TGI) of 95.1%. Similarly, hz1E11 as a single agent had similar *in vivo* antitumor activity to trastuzumab (S1 Fig) and the combination of hz1E11 with trastuzumab also showed dose-dependent anti-tumor activity in the NCI-N87 xenograft mouse model (Fig 2D). Antibody combination treatment at 1 mg/kg each of hz1E11 and trastuzumab resulted in similar anti-tumor activity with trastuzumab single treatment at 10 mg/kg, and at the 2.5 mg/kg combination and above the established tumor stabilized or started regressing (No statistically significant difference [*P* < 0.05] was observed among 2.5, 5, and 10 mg/kg combinations). These results show that hz1E11 has the affinity and biological activity equivalent to ch1E11, and that hz1E11 enhances the anti-tumor activity of trastuzumab when used in combination.

### Alanine scanning of CDR-H3 and CDR-L3

**Fig 3:**
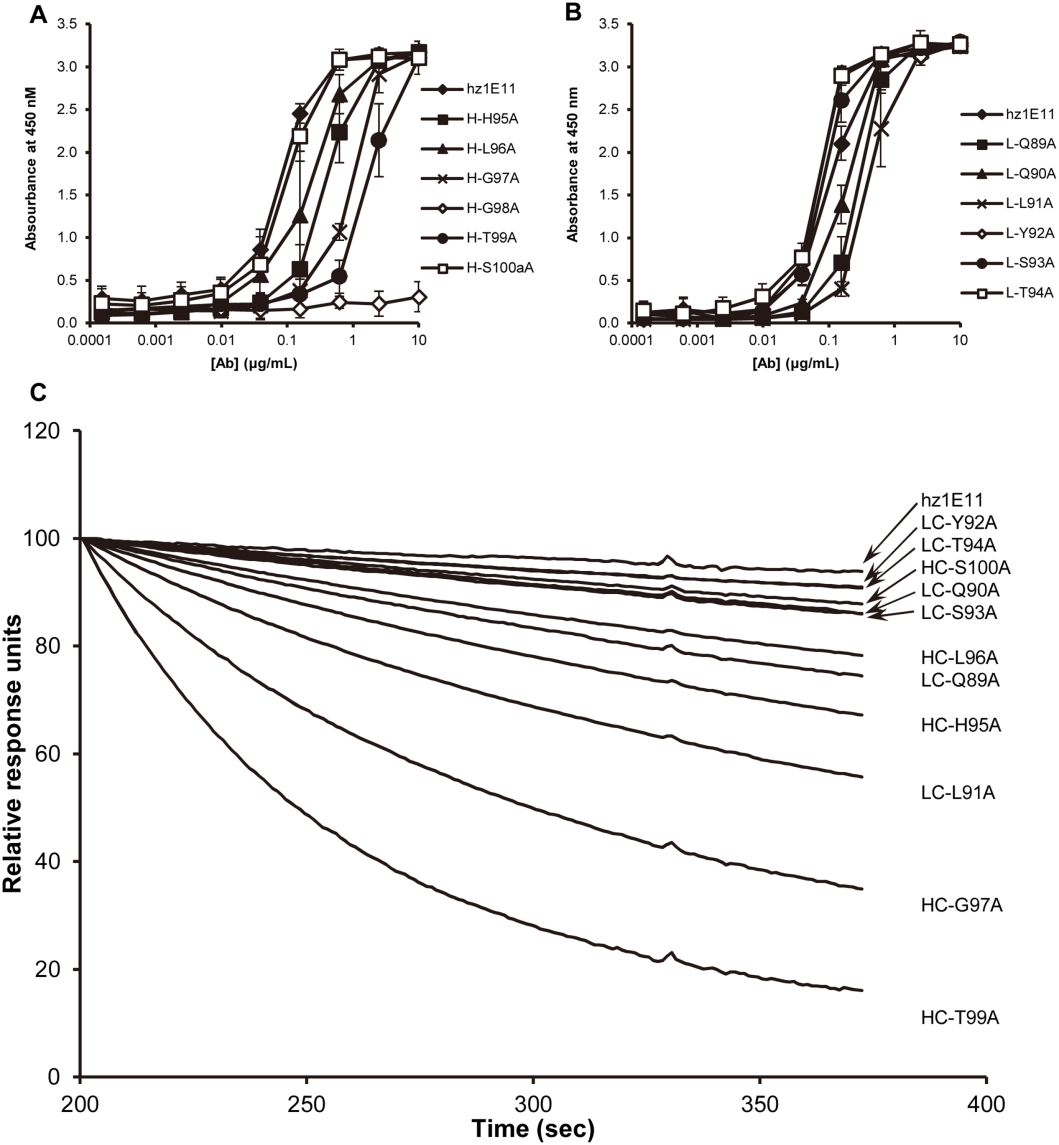
Three residues in CDR-H3 are important for antigen-antibody binding. The binding activities of alanine scanning mutants of CDR-H3 (**A**) and CDR-L3 (**B**) were analyzed by ELISA. **C**, Quantitative dissociation kinetics of indicated mutants were analyzed by surface plasmon resonance. HER2-ECD protein was immobilized on a CM5 sensor chip followed by exposure to indicated mutant Fab antibodies. The 100% association was defined as the response unit (RU) at 200 seconds from the beginning of Fab injection. *k*_off_ values were calculated using the BIAevaluation software.

To identify the critical residues for antigen-antibody interaction, alanine scanning mutagenesis was carried out in CDR3 regions of heavy and light chains (CDR-H3 and CDR-L3). The effects of the mutations were assessed by analyzing the binding activity of purified Fab proteins by indirect ELISA. In CDR-H3, alanine substitution at position Gly98_H_ abolished the binding of the Fab to HER2, and G97_H_A and T99_H_A mutants showed reduced binding activity (Fig 3A). In CDR-L3, the alanine substitutions had much smaller effects (Fig 3B). The *k*_off_ values of alanine scanning mutants were analyzed using SPR against immbolized HER2-ECD protein. We confirmed that the heavy chain G98A mutant completely lost binding activities, and the neighboring T99_H_A and G97_H_A mutants resulted in 32-fold and 17-fold increased *k*_off_ values, respectively (Fig 3C). These results indicate that heavy chain Gly98_H_ is functionally critical and its adjacent residues are also functionally important for antigen-antibody interaction.

### Affinity maturation of hz1E11

**Fig 4:**
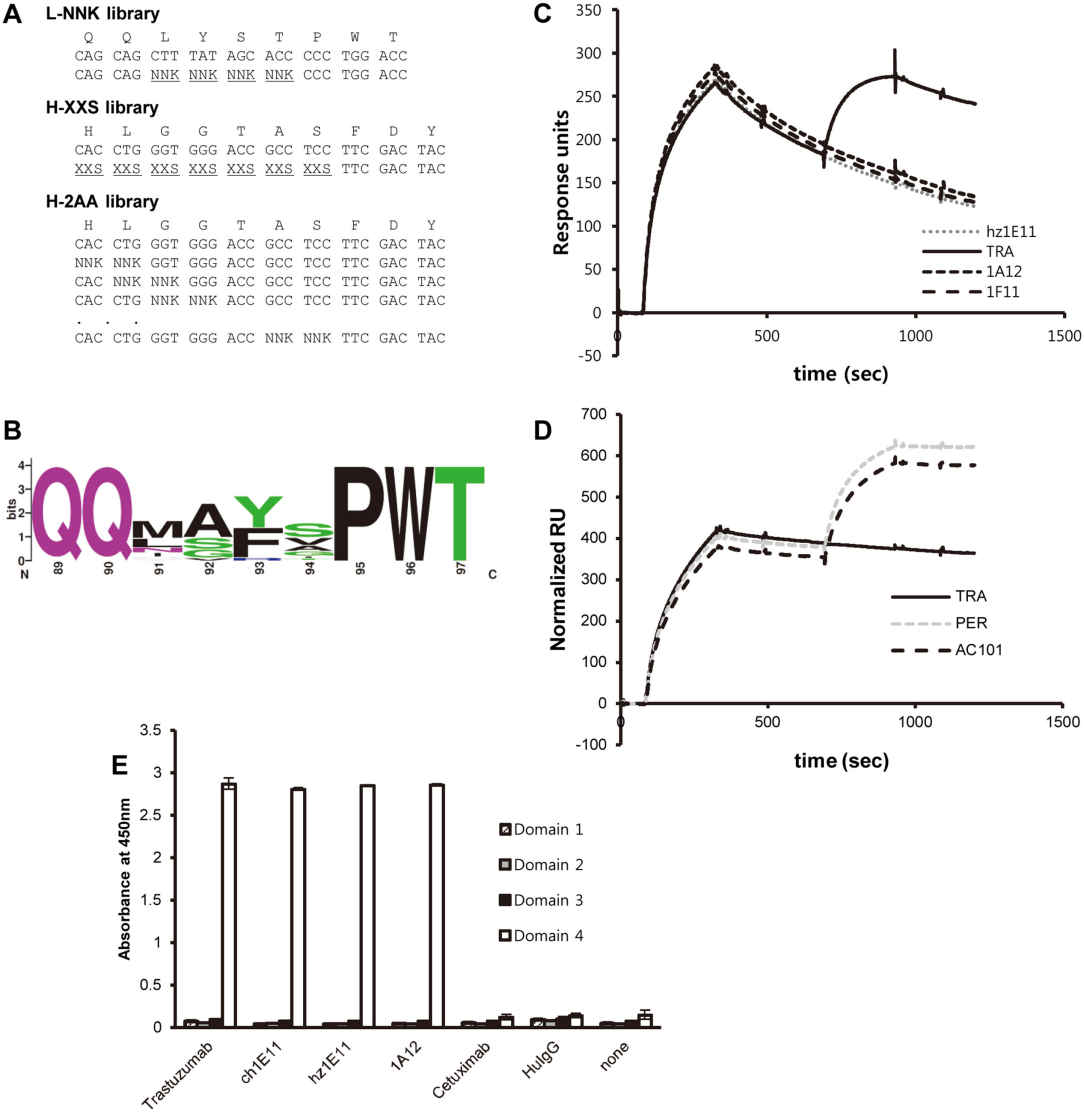
Library design, panning and binding properties of affinity-matured clones. **A**, Design of the CDR-L3 and CDR-H3 libraries for affinity maturation. See text for detailed explanation. **B**, Sequence logo was generated using WebLogo program about unique CDR-L3 sequences from 4^th^ round panning-2 (low-stringency panning) output of the CDR-L3 library (*n* = 39). **C**, The hz1E11 was immobilized on a CM5 sensor chip followed by sequential exposure to HER2-ECD-His and indicated antibodies using BIAcore2000. **D**, Trastuzumab was immobilized on a CM5 sensor chip followed by sequential exposeure to HER2-ECD-His and indicated antibodies using BIAcore 3000. **E**, The binding activities of the antibodies were analyzed by ELISA using recombinant HER2 sub-domain proteins.

Affinity maturation of hz1E11 was conducted by the construction of heavy or light chain CDR3 variant libraries, followed by the equilibrium selection of the phage antibody libraries. For the light chain, glutamine residues at positions 89_L_ and 90_L_ were not diversified because glutamine is commonly found at these positions of nine amino acid-longCDR-L3 sequences at 59% and 73% frequencies, respectively [27]. Positions 91_L_–94_L_ were randomized using a NNK degenerate codon (L-NNK). For the heavy chain, positions 95_H_ -100a_H_ were randomized using a XXS codon (H-XXS), designed so that at the first and second nucleotide positions of the diversified codon the wild type base occurred with 70% chance, each of the other three bases occurred at 10% frequency, and the third letters of the codons were synthesized using an equimolar mixture of G and C (Fig 4A). High affinity binders were selected from high-stringency or low-stringency panning (Table 1).

**Table 1:** Panning results of CDR-L3 and–H3 libraries.

		ELISA positive ratio				Diversity	
Library	Strategy	1^st^ output	2^nd^ output	3^rd^ output	4^th^ output	Output	CDR-L3 OR–H3 (colonies/tested)
L-NNK	Panning-1 ^1)^	73%	80%	33%	0%	3^rd^ output	QQNAYAPWT (15/34) QQLGFIPWT (10/34) QQNAFSPWT (2/34)
	Panning-2 ^2)^	80%	100%	100%	100%	4^th^ output ^3)^	QQTAFSPWT (4/55) QQIAYVPWT (3/55) QQMSYGPWT (3/55) QQMSYVPWT (3/55)
H-XXS	Panning-1	73%	50%	20%	3%	2^nd^ output	NYGGTASFDY (8/9) HFGGTASFDY (1/9)
	Panning-2	87%	100%	100%	100%	4^th^ output	NFGGTASFDY (3/5) HFGGTASFDY (1/5) HLGGTASFDY (1/5)
H-2AA	Panning-1	73%	30%	33%	10%	2^nd^ output	NYGGTASFDY (5/7) NLGGTASFDY (2/7)
	Panning-2	73%	97%	100%	100%	4^th^ output	NFGGTASFDY (6/10) NYGGTASFDY (2/10) HWGGTASFDY (1/10) HYGGTASFDY (1/10)

^1)^ In panning-1 (high stringency panning), the starting concentration of panning antigen in the first round is 100 pM, then reduce successively by 10-fold in each subsequent round.

^2)^ In panning-2 (low stringency panning), the concentration of panning antigens for 1^st^, 2^nd^, 3^rd^, and 4^th^ rounds are 100 nM, 1 nM, 100 pM, and 10 pM, respectively.

^3)^ Due to the diversity of the panning output of L-NNK library from panning-2, only 4 highest enriched clones are presented. Substituted amino acids are underlined.

Compared to the low-stringency panning of the L-NNK library in which 100% of the second-, third-, and fourth-round output clones screened were ELISA positive, for the high-stringency panning the fourth-round output yielded no binder at all, and only one-third of the third-round output clones were ELISA positive (Table 1). The selected clones from the CDR-L3 library included many sequences that had all four randomized CDR-L3 positions mutated, unlike CDR-H3 libraries from which most of the selected clones retained the wild type sequence in positions 97_H_ -100_H_ (see below). Different panning strategies yielded different sequence enrichment patterns. For example, the light chain variant clone 1A12 (Q_89L_QNAYAPWT_97L_) was isolated from the high-stringency panning but not from the low-stringency panning, and the heavy chain variant clone 1B12 (N_95H_YGGTASFDY_102H_) was also selected only from the high-stringency panning. Interestingly, 59% of unique antibodies in 4^th^ output with low-stringency panning of L-NNK library had an alanine at position 92_L_, in line with alanine scanning analysis (Fig 4B).

Almost all clones that were isolated from the H-XXS library had the same sequence at 97_H_ –100_H_ as the parental clone, and showed a clear enrichment of Asn-Tyr sequence at 95–96. To further assess the contribution of CDR-H3 residues to antigen-antibody binding, an additional CDR-H3 library was constructed with twin NNK random codon scanning of CDR-H3 (H-2AA, Fig 4A). In low-stringency panning, mutants at positions 95_H_, 96_H_, 99_H_, 100_H_, and 100a_H_ were selected in the first-round panning, but only mutants at positions 95_H_ and 96_H_ were enriched in the fourth-round output (Table 1). We could not detect the mutants at positions Gly97_H_ and Gly98_H_ in any panning strategies and outputs.

### Affinity and binding activity of selected clones

To assess the combination effect of the affinity-matured heavy and light chain variants, IgG antibodies with combinations of affinity-matured heavy and light chains were produced and their *k*_off_ values were determine using SPR analysis (Table 2). Among two light chain variants and two heavy chain variants, 1A12 derived from L-NNK showed the greatest improvement (16-fold). The combination of the light chain of 1A12 with the optimized heavy chain of the clone 1B12 resulted in a 24-fold improvement in *k*_off_ over the parental hz1E11, while for the other combinations the *k*_off_ values were similar to or larger than that of 1A12. Because the 1.5-fold additional improvement by the L-1A12 + H-1B12 combination was not significantly large, clones 1A12 and 1F11 (light chain variants) were chosen for further characterization to minimize the sequence difference of the affinity-matured antibodies from the parental clone. The dissociation constants for the affinity matured clones, also determined by SPR analysis, were more than 10-fold lower than that of the parental clone hz1E11 and comparable to that of trastuzumab (Table 3).

**Table 2:** *k*_off_ measurement of selected clones.

CLONE	CDR-L3 sequences^1)^	CDR-H3 sequences^1)^	*k*_off_ (sec^-1^)
hz1E11	QQLYSTPWT	HLGGTASFDY	4.48E-4
L-1A12	QQNAYAPWT	HLGGTASFDY	2.80E-5
L-1F11	QQTAFSPWT	HLGGTASFDY	5.89E-5
H-1B12	QQLYSTPWT	NYGGTASFDY	7.40E-5
H-2A7	QQLYSTPWT	NFGGTASFDY	5.20E-5
L-1A12 + H-1B12	QQNAYAPWT	NYGGTASFDY	1.87E-5
L-1F11 + H-1B12	QQTAFSPWT	NYGGTASFDY	3.45E-5
L-1A12 + H-2A7	QQNAYAPWT	NFGGTASFDY	2.59E-5
L-1F11 + H-2A7	QQTAFSPWT	NFGGTASFDY	4.50E-5

^1)^ Substituted amino acids are underlined.

**Table 3:** Binding kinetics of the anti-HER2 antibodies. ^1)^

Clone	*K*_D_ (nM)	*k*_a_ (M^-1^s^-1^)	*k*_d_ (s^-1^)	R_max_	χ^2^
Trastuzumab	3.2	5.2×10^4^	1.7×10^-4^	42.2	4.26
hz1E11	23	3.5×10^4^	8.2×10^-4^	62.3	2.14
1A12	1.9	6.4×10^4^	1.2×10^-4^	69.4	5.62
1F11	1.4	9.0×10^4^	1.2×10^-4^	64.4	6.14

^1)^ The kinetic parameters were obtained by fitting the binding data to 1:1 (Langmuir) binding model.

To determine whether affinity-matured hz1E11 clones bind to the same epitope as the parental hz1E11, the binding of 1A12 and 1F11 to the HER2-ECD that was pre-bound to hz1E11 was analyzed by SPR. Both clones were unable to bind to the monomeric HER2-ECD-His protein captured by immobilized 1E11 whereas trastuzumab could (Fig 4C). Additionally, it was confirmed that the 1A12 epitope does not overlap with that of trastuzumab (Fig 4D). Clones 1A12 and 1F11 also bind to sub-domain IV like their parental clone hz1E11 (Fig 4E). These data indicate that epitopes of 1A12 and 1F11 were not changed by the affinity maturation process.

### Efficacy of affinity matured hz1E11 clones

**Fig 5:**
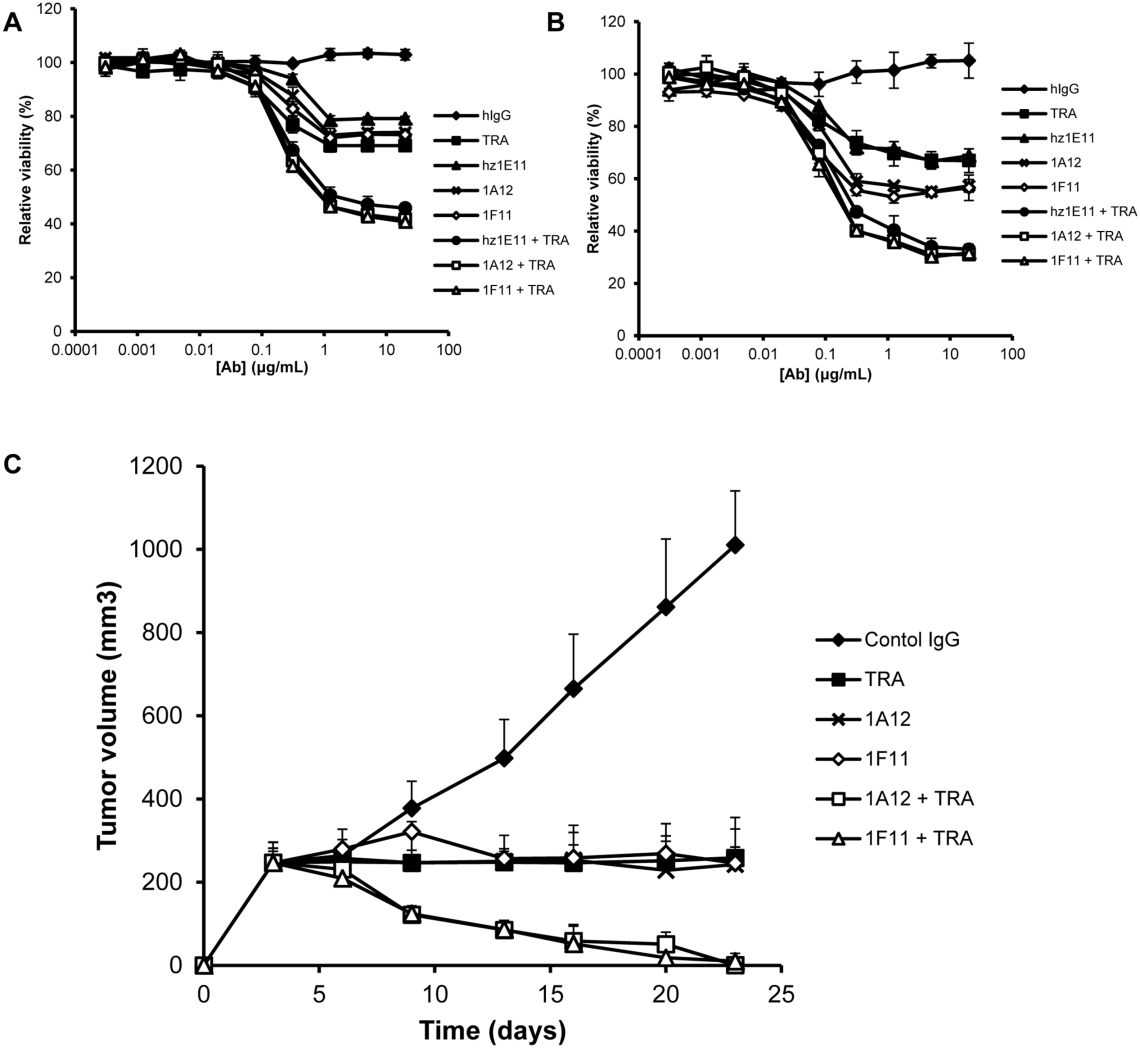
Affinity-matured hz1E11 clones shows antitumor activity in HER2-overexpressing gastric cancer models. NCI-N87 cells (**A**) and OE-19 cells (**B**) were treated with antibodies for 4 days in the complete growth media. The cell viability was measured in duplicates (mean ± SD) using WST-1 reagent. The 100% viability was defined as the viability of the antibody-untreated wells. **C**, NCI-N87 (*n* = 5 mice/group) cells were inoculated into mice and antibody treatments started when tumor volumes reached approximately 200 mm^3^. Mice received a dose of 20 mg/kg for single agent treatment and 10 mg/kg of each antibody for combination treatment. Administration days are indicated by arrows. Tumor volume (mm^3^) was expressed as the mean ± SD. For clarity, only positive error bars are shown.

Both 1A12 and 1F11 antibodies showed slightly increased anti-proliferative activities as a single agent compared to hz1E11 on NCI-N87 and OE-19 gastric cancer cell lines that overexpress HER2 (Fig 5A and 5B), whereas in combination with trastuzumab, their anti-proliferative activities was superior to that trastuzumab alone and equivalent to hz1E11 plus trastuzumab. The anti-proliferative activity of 1A12 and 1F11 was confirmed *in vivo* in NCI-N87 xenograft model. Superior antitumor activity was evident in combination with trastuzumab to that of each agent alone in both xenograft models (Fig 5C).

## Discussion

Despite encouraging clinical results obtained with the HER2 targeting antibody trastuzumab in the treatment of gastric cancer, there still are needs for more potent HER2 targeted therapies [6]. Combination of non-competing antibodies targeting receptors, such as HER2, EGFR, and VEGFR3, can increase anti-tumor activity in preclinical models [11–13, 28]. Pertuzumab, another HER2-targeting antibody, is approved for use in combination with trastuzumab in the treatment of metastatic breast cancer [29]. In a previous study, we developed a novel HER2 targeting antibody, 1E11, which was demonstrated to have significant anti-tumor activity as a single agent and synergistic effect in combination with trastuzumab *in vitro* and *in vivo* [22]. The anti-tumor activity of the 1E11 plus trastuzumab combination is superior not only to trastuzumab single treatment but also to the combination of pertuzumab and trastuzumab. In this report, the humanization and subsequent affinity maturation of the mouse hybridoma-derived 1E11 monoclonal antibody is described.

Initial approaches to reduce potential immunogenicity of nonhuman variable regions by CDR-grafting into a human framework significantly decrease the immunogenicity of therapeutic antibodies [15, 17]. Superhumanization using human germline frameworks as template has been proposed as a superior humanization methodology to avoid putative effector T-cell epitopes derived by somatic hypermuations of human framework [30–32]. It has been suggested that antibodies encoded by germline gene segments are structurally flexible and able to accommodate binding to many different antigens [33–35]. The CDRs of murine antibody 1E11 as well as one residue in the Vernier zone of V_H_ (Ala49_H_) were grafted onto the human germline variable and joining genes with highest homology to the parental antibody (Fig 1). The resulting humanized antibody, hz1E11, showed almost identical affinity and biological activity to the parental antibody (Fig 2).

Selection of residues for randomization is a critical step in targeted randomization approach of affinity maturation because of the practical limitations of antibody library size and the selection technologies. Alanine scanning and *in silico* analysis of the antibody sequence, especially in CDRs, are useful for selecting the randomization target residues and facilitate the affinity maturation process. In alanine scanning analysis of CDR-H3 of hz1E11, the G98A mutant completely lost binding activity and the G97A mutant showed reduced binding activity (Fig 3A). Direct alteration of an essential paratope residue usually results in the total loss of binding ability of the antibody [26, 33, 36]. Therefore, a more conservative approach to CDR-H3 randomization was employed, using either a hand-mixed oligonucleotide that is biased toward the parental sequence, or the twin NNS random codon scanning of CDR-H3. As expected from the alanine scanning analysis, almost all clones that were isolated from the CDR-H3 libraries had the sequence 97_H_–100a_H_ same as the parental clone (G_97H_GTAS_100aH_).

The alanine scanning results suggest that all CDR-L3 residues are dispensable, although some of the mutations seems to lower the affinity (Fig 3B). Therefore, four positions of CDR-L3 (91_L_–94_L_) were fully randomized using the NNK degenerate codon. The sequence analysis of the selected clones confirmed the alanine scanning analysis; the majority of the selected clones from the CDR-L3 library had all four randomized CDR-L3 positions changed from the parental residue, contrary to the CDR-H3 optimization results in which most of the selected clones from the libraries had the same sequence as the parental hz1E11 in the middle part of the CDR (Table 1). The clones with most affinity improvement came from the CDR-L3 library. It is probable that the CDR-H3 of hz1E11 is already close to optimal and most mutations, especially in the region 97_H_–100_H_, are deleterious to the binding affinity, while the CDR-L3 sequence is not as optimal and the mutation in this region can result in significant improvement in the affinity. The L-NNK library panning outputs were enriched with clones with a hydrophobic amino acid at the position 91_L_, a small amino acid at 92_L_, and an aromatic amino acid at 93_L_. This pattern is somewhat different from the parental CDR-L3 sequence of Q_89L_QLYSTPWT_97L_ and again suggests that the CDR-L3 sequence of the parental 1E11 antibody was sub-optimal and thus had room for affinity improvement.

The clones with most improvement in affinity, 1A12 and 1F11, came from the high-stringency panning of the CDR-L3 NNK library, although 1F11 was also found from the low-stringency panning of the same library (Table 2). Compared to the low-stringency panning in which 100% of the second-, third-, and fourth-round output clones screened were ELISA positive, for the high-stringency panning the fourth-round output yielded no binder at all, and only one-third of the third-round output clones were ELISA positive (Table 1). It is likely that the extremely antigen-limiting condition favored clones with higher affinity. However, at the same time, the basal level of non-specific binding of phage particles became more evident, and even dominated the panning output. In agreement with this argument, sequence analysis of the ELISA positive clones showed that the high-stringency panning resulted in the convergence of the output clones to a small number of sequences, while the sequences were more divergent for the output clones from the low-stringency panning. For the CDR-H3 libraries, the overall pattern of the panning output titer and the ratio of ELISA positive clones were similar to that of the CDR-L3 libraries; i.e. higher panning stringency had a negative effect on both parameters. Not surprisingly, the highly enriched clone (1B12) with CDR-H3 sequence N_95H_YGGTAS_100aH_ had *k*_off_ value that was more than 5 times slower than that of the parental antibody. The combination of the V_H_ of 1B12 and V_L_ of 1A12 further improved *k*_off_ and *K*_D_ slightly.

The humanized, affinity matured anti-HER2 clones showed inhibitory activity on the growth of HER2-positive gastric cancer cell lines *in vitro* and *in vivo* xenograft models (Figs 2 and 5). The inhibitory activity did not reflect the improvement in the binding affinity, however, and their maximal growth inhibition and IC_50_ values were comparable to those of the parental hz1E11, possibly because the hz1E11 already had sufficiently high inhibitory activity on HER2-overexressing cancer cell lines. This does not necessarily mean that they would have similar clinical efficacies, and the effect of the affinity improvement of hz1E11 on the growth of HER2-positive human tumors needs to be evaluated in future development research. The *in vivo* efficacy may also be enhanced by higher antigen-binding affinity. The affinity of 1A12 can be further improved by e.g. the diversification of the CDRs other than CDR-H3 and CDR-L3, or selection of optimal CDR-H3 sequence in the context of 1A12 light chain sequence.

To summarize, an anti-HER2 murine antibody, 1E11, was humanized and affinity matured through extensive library construction, panning, and screening efforts. High-stringency, antigen-limiting selection conditions facilitated the isolation of affinity matured clones, among which the best ones were CDR-L3 variants. Different CDR diversification strategies produced different sequence enrichment patterns, with varying degrees of success in affinity improvement. On the other hand, the stringency of panning had similar effects on the output pattern for all the libraries. It is anticipated that these results can be utilized to improve the experimental design of future affinity maturation efforts and generate highly optimized antibody clones for therapeutic and other applications.

## Supporting Information

S1 Fig *In vivo* antitumor activity of hz1E11. Mice bearing NCI-N87 xenograft tumors were treated with 10 mg/kg of control antibody, trastuzumab, hz1E11, or trastuzumab plus hz1E11. Palivizumab was used as the isotype control antibody. Tumor volume (mm^3^) was expressed as mean ± SD (*n* = 6 mice/group).
(TIF)
