# Supplementary figures and images for: Affinity Maturation of Monoclonal Antibody 1E11 by Targeted Randomization in CDR3 Regions Optimizes Therapeutic Antibody Targeting of HER2-Positive Gastric Cancer
Source: PLoS One. 2015 Jul 30;10(7):e0134600. doi: 10.1371/journal.pone.0134600 (PMC4520604; doi:10.1371/journal.pone.0134600)

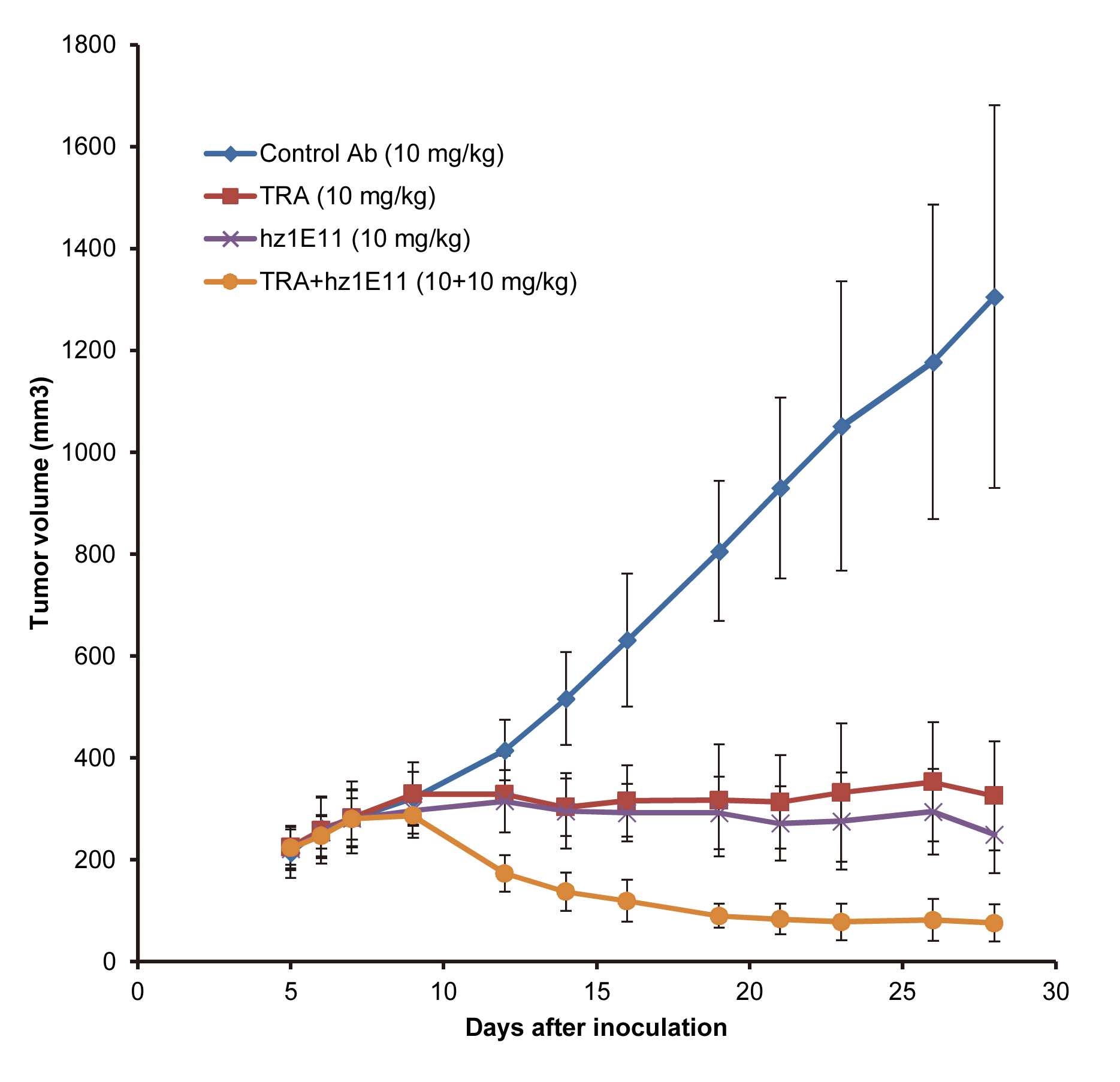

Supplement: S1 Fig — Mice bearing NCI-N87 xenograft tumors were treated with 10 mg/kg of control antibody, trastuzumab, hz1E11, or trastuzumab plus hz1E11. Palivizumab was used as the isotype control antibody. Tumor volume (mm3) was expressed as mean ± SD (n = 6 mice/group). (TIF) [file pone.0134600.s001.tif]
